# Computed tomography dose index and dose length product for cone‐beam CT: Monte Carlo simulations of a commercial system

**DOI:** 10.1120/jacmp.v12i2.3395

**Published:** 2011-01-19

**Authors:** Sangroh Kim, Haijun Song, Ehsan Samei, Fang‐Fang Yin, Terry T. Yoshizumi

**Affiliations:** ^1^ Medical Physics Graduate Program Duke University Medical Center Durham NC 27710 USA; ^2^ Department of Radiation Oncology Duke University Medical Center Durham NC 27710 USA; ^3^ Carl E. Ravin Advanced Imaging Laboratories Duke University Medical Center Durham NC 27710 USA; ^4^ Duke Radiation Dosimetry Laboratory Duke University Medical Center Durham NC 27710 USA

**Keywords:** CBCT, CT dose index, dose length product, BEAMnrc, Monte Carlo

## Abstract

Dosimetry in kilovoltage cone beam computed tomography (CBCT) is a challenge due to the limitation of physical measurements. To address this, we used a Monte Carlo (MC) method to estimate the CT dose index (CTDI) and the dose length product (DLP) for a commercial CBCT system. As Dixon and Boone^(1)^ showed that CTDI concept can be applicable to both CBCT and conventional CT, we evaluated weighted CT dose index $\left({\text{CTDI}}_{w}\right)$ and DLP for a commercial CBCT system. Two extended CT phantoms were created in our BEAMnrc/EGSnrc MC system. Before the simulations, the beam collimation of a Varian On‐Board Imager (OBI) system was measured with radiochromic films (model: XR‐QA). The MC model of the OBI X‐ray tube, validated in a previous study, was used to acquire the phase space files of the full‐fan and half‐fan cone beams. Then, DOSXYZnrc user code simulated a total of 20 CBCT scans for the nominal beam widths from 1 cm to 10 cm. After the simulations, CBCT dose profiles at center and peripheral locations were extracted and integrated (dose profile integral, DPI) to calculate the CTDI per each beam width. The weighted cone‐beam CTDI $\left({\text{CTDI}}_{w,l}\right)$ was calculated from DPI values and mean ${\text{CTDI}}_{w,l}\;\bar{\left({\text{CTDI}}_{w,l}\right)}$ and DLP were derived. We also evaluated the differences of ${\text{CTDI}}_{w}$ values between MC simulations and point dose measurements using standard CT phantoms. In results, it was found that $\bar{{\text{CTDI}}_{w,600}}$ was 8.74±0.01 cGy for head and $\bar{{\text{CTDI}}_{w,900}}$ was 4.26±0.01 cGy for body scan. The DLP was found to be proportional to the beam collimation. We also found that the point dose measurements with standard CT phantoms can estimate the CTDI within 3% difference compared to the full integrated CTDI from the MC method. This study showed the usability of CTDI as a dose index and DLP as a total dose descriptor in CBCT scans.

PACS number: 87.57.uq

## I. INTRODUCTION

The computed tomography dose index (CTDI), originally proposed by Jucius and Kambic^(2)^ and established by Shope et al.,^(3)^ has served as a standard measure of radiation dose in CT since the 1980s. Although there are a few variants in CTDI such as ${\text{CTDI}}_{\text{FDA}},{\text{CTDI}}_{100},{\text{CTDI}}_{300}$ and ${\text{CTDI}}_{\infty },$ ^(^ ^3^ ^–^ ^7^ ^)^ these are conceptually equivalent to the original definition of CTDI by Shope et al., except for the range of measurements. Due to operational simplicity of the measurements and availability of the standardized 100 mm length pencil ion chamber, ${\text{CTDI}}_{100}$ has been generally accepted as a standard CT dose descriptor^(5)^ that can be expressed as follows:

(1) $$CTDI_{100}=\frac{1}{nT}\int_{-50mm}^{50mm}D(z)dz$$

where *n* equals number of slices, *T* equals slice thickness, and *D(z)* equals dose profile along the axis of rotation.

In recent years, the CT technology has advanced resulting in substantially wider beam width; this has enabled the acquisition of a larger imaging area with fewer numbers of rotations and shorter scan time. Typical cone‐beam CT (CBCT) systems perform a single X‐ray tube rotation with a stationary table to acquire a three‐dimensional (3D) image. Despite all the benefits of the wide beam CBCT system, this beam geometry created the difficulty of estimating patient dose because conventional CTDI dosimetry is no longer applicable. In addition, the cone‐beam creates an extended range of the axial dose profile (typically 30–100 cm) beyond the regular 100 mm pencil ion chamber, resulting in long tail portions of ionization which can not be fully collected with the conventional pencil ion chamber. Although investigators previously reported the use of an extended 300 mm long pencil ion chamber,^(^ ^6^ ^,^ ^8^ ^)^ the chamber needs to have the accuracy related to electron collection efficiency, sensitivity variation and stem leakage.^(9)^ Additionally, this requires CT phantoms with extended length.

A volume of research has been performed to evaluate the limitation of direct application of CTDI in CBCT dosimetry. Mori et al.^(8)^ studied the beam width effect on a 256‐slice CT and found that if the beam width is over 20 mm, the length of body phantom needs to be larger than 300 mm to collect > 90% of dose profile integral (DPI). Boone^(7)^ found from his Monte Carlo (MC) simulations that the use of ${\text{CTDI}}_{100}$ even for 10 mm of slice thickness was not efficient. Geleijns et al.^(6)^ introduced a pragmatic metric named average absorbed dose within pencil ion chamber, ${\bar{D}}_{100}$ to characterize the CTDI and compared it to ${\text{CTDI}}_{300}$ for a 320‐slice CT scanner. In their study, they defined the ${\text{CTDI}}_{100}$ and ${\text{CTDI}}_{300}$ as follows:

(2) $${\bar{D}}_{100}=\frac{1}{100}\int_{-50mm}^{50mm}D(z)dz=\frac{nT}{100}CTDI_{100}$$

(3) $$CTDI_{300}=\frac{1}{nT}\int_{-150mm}^{150mm}D(z)dz$$

They showed excellent agreements between ${\text{CTDI}}_{300\text{air}}$ and ${\text{CTDI}}_{100}\text{air}$, while ${\text{CTDI}}_{100\text{air}}$ substantially underestimates ${\text{CTDI}}_{300\text{air}}$. (Note that the subscript air represents free‐in‐air measurement.) They also found the same results in their MC simulations, which used a 700 mm long CT body phantom. Their conclusion was that 350 mm long CT phantoms would be adequate to obtain a reasonable CTDI for the 320‐slice CT scanner. However, the use of such a long phantom creates a practical handling problem (a 350 mm long body phantom weight is about 34.5 kg), as well as a higher manufacturing expense. To avoid these limitations, Dixon et al.^(^ ^10^ ^,^ ^11^ ^)^ suggested an alternative method to estimate CTDI by measuring a point dose with a small ion chamber in helical CT scan mode and validated it in MDCT scanner.

Dixon et al. introduced the CTDI‐aperture $\left({\text{CTDI}}_{a}\right)$ concept and verified its constancy to within a few percent for a GE LightSpeed 16‐slice CT scanner. Mori et al.^(8)^ also showed that DPI for the 256‐slice CT scanner was linearly proportional to the beam width that implies the constancy of ${\text{CTDI}}_{a}$. The ${\text{CTDI}}_{a}$ (equivalent to “equilibrium dose parameter” $A_{\text{eq}}$ in Dixon and Boone's paper^(1)^ ^)^ is defined as follows:

(4) $$CTDI_{a}=A_{eq}=\frac{1}{a}\int_{-\infty }^{\infty }D(z)dz,=\text{constant}$$

where *a* equals actual size of beam aperture and *D(z)* equals dose profile along the axis of rotation.

**Figure 1:**
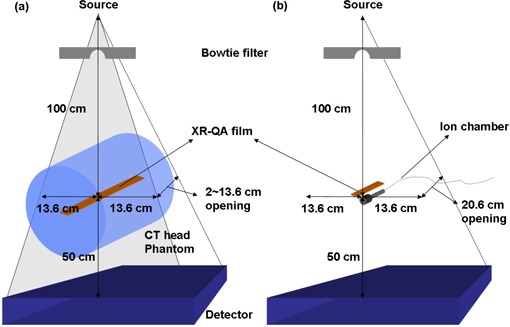
Radiochromic film measurement setups for various CBCT beam widths: (a) axial dose profile measurements with film strips; (b) film calibration with an ion chamber.

One should note the fundamental difference in scan geometry between helical CT and the single rotation CBCT. The point dose accumulated in the helical CT is the integral dose along the longitudinal direction with continuous couch movement, while that of CBCT is a dose accumulated from several projection shots with a single rotation. Therefore, the irradiated fluence of the helical CT beam is relatively uniform along the longitudinal direction, whereas that of CBCT beam is nonuniform due to large scatters and heel effect. However, Dixon and Boone^(1)^ found that cone beam profile can be decomposed as a group of several narrow beam profiles that showed a unified dosimetric approach in all CT scan modalities. (See Fig .1. Dixon and Boone^(1)^)

In addition, Dixon and Boone^(1)^ provided the correlation between the central ray dose, f(0) and $A_{\text{eq}}$ in CBCT by using analytic convolution method with Monte Carlo data. However, due to the freshness of the concept, there is no dosimetric data published using this concept for a commercial CBCT system. In addition, there is no method suggested to estimate the total dose (DLP) in CBCT scan which could be used to derive the effective dose (ED) for a CBCT scan.

In this study, we adopted Dixon's ${\text{CTDI}}_{a}$ concepts to estimate the CTDI and DLP values for a commercial CBCT system. The CTDI was estimated by using an On‐Board Imager for head and body CBCT scans with various beam widths. An MC technique with extended CT phantoms was used to overcome the limitation of the pencil ion chamber CTDI measurements. We also evaluated the CTDI differences between MC simulations (full integration in the extended 60 and 90 cm long phantom) and point dose measurements (central ray dose, f(0)) using 15.2 cm long standard CT phantoms. This allowed us to evaluate the accuracy of the point dose measurements in standard phantoms that could be a clinically feasible dosimetry method in CBCT.

## II. MATERIALS AND METHODS

We employed an On‐Board Imager (OBI, Varian Medical Systems, Palo Alto, CA) to investigate the CTDI dosimetry in cone beam geometry. An MC model of the OBI system was used to simulate two types of the CBCT scan: full‐fan and half‐fan modes. Full‐fan CBCT scan was used for the head CT phantom, while half‐fan CBCT scan was performed for the body CT phantom. (The detailed information of the CBCT scan modes can be found in Yoo et al.^(12)^) The axial dose profiles for the nominal beam openings from 1 cm to 10 cm specified at isocenter, were obtained from the MC simulations, and CTDI and DLP were calculated using the profile data. Before the simulation, we verified the axial dose profiles with radiochromic film.

### A. Measurements of CBCT beam width and profiles

To check the size of actual beam widths and the relative beam profile of a CBCT scan, the axial dose profiles of CBCT beams were measured with a radiochromic film (Model GafChromic XR‐QA, International Specialty Products, Wayne, NJ) both free‐in‐air and in a CT phantom. The experimental setup of the axial profile measurements in phantom is shown in Fig. 1(a).

First, we placed a film strip (17 cm×1 cm) free‐in‐air to verify the beam opening parameters of the OBI system console. After this verification, a new film strip was placed inside a head CT phantom (diameter=16 cm,length=15 cm; CIRS, Norfolk, VA) to avoid the difficulties in MC simulations of film response free‐in‐air. It is challenging to estimate an accurate dose response for a small dimension detector such as thin film due to the low probability of photon interactions for the small cross‐section of the detector. Although embedding the film into the phantom make MC simulation feasible, this setup could generate large scatters inside the phantom that broaden the beam profile. But note that the purpose of this film profile measurement was to validate the MC model, not to generate the accurate beam profile shape of the CBCT system. With these measurement settings, a CBCT scan was performed with the following settings: full‐fan mode, 125 kVp, 80 mA, pulsed 25 ms, 360° rotation with 660 projections using the OBI system. One strip of the film was exposed for each of the CBCT beam width settings: 1 to 10 cm of nominal beam widths per 1 cm step. It should be clearly noticed that only the head CT phantom was employed in this measurement. We found that 125 kVp, 80 mA cone beam could not expose enough radiation (without overheating the X‐ray tube) to the film strip in the body CT phantom due to the large thickness of the phantom.

The net optical density (NOD) to dose calibration for the film was performed on the CBCT beams. As can be seen in Fig. 1(b), a 6 cm^3^ ion chamber (Model: 10×5−6, Radcal, Monrovia, CA) and a strip of the film were placed in parallel at the isocenter of the CBCT system and irradiated from a stationary CBCT beam by varying the exposure range from 0 to 16.07 R. All the exposed film strips (both calibration and axial dose profile strips) were placed in a dark place for approximately one day to allow full development. A high‐resolution flatbed scanner (Model: Perfection 4990 Photo, Epson, Long Beach, CA) was used for the film digitization. The film strips were scanned in a reflective, red‐green‐blue (RGB) mode (16 bit per color), and 72 dot per inch (dpi) resolution with no color correction and the results were saved as in TIFF image file format. The image files were imported into MATLAB software to convert the pixel values (PV) of the film images into exposure (R). Since the absorption spectrum of the radiochromic film shows a maximum sensitivity for the red light (as previously studied by Stevens et al.^(13)^), only the PVs of red channel were used in the film dosimetry. To improve the statistics of the PVs, a region of interest (ROI) was drawn for each calibration image with an area of ~ 500 pixels and the mean value for the ROI was used as a representative of the PVs. The exposure values of the ion chamber were converted into the absorbed dose by multiplying the roentgen‐to‐rad conversion factor (0.869). The conversion factor from dose‐to‐air to dose‐to‐film was 1.00. The axial profile of the various beam width was extracted from the digitized film images, normalized and compared to the MC results in order to verify the MC model of the CBCT system. As with the film measurements, the MC simulation was performed with a standard head CT phantom (15.2 cm in length). A one‐dimensional (1D) bicubic interpolation method was used to convert the PVs into the exposures using the calibration data.

### B. Monte Carlo simulations

A Monte Carlo model of a Varian OBI X‐ray tube, developed in the previous study,^(14)^ was employed to simulate the CBCT scans. (Details of the X‐ray tube model can be found in the previous paper.^(14)^) Using the model, CBCT scans were simulated for the actual CBCT beam widths of the OBI system in the BEAMnrc/EGSnrc MC system.^(^ ^15^ ^,^ ^16^ ^)^ Beam parameters for the CBCT scans were as follows: ISOURCE=10 (parallel circular beam incident from side), incident electron energy=125 keV, beam diameter=0.6 cm.

**Figure 2:**
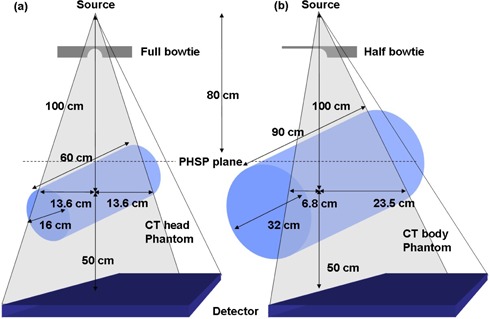
CBCT irradiation setups with extended CT phantoms simulated in the MC systems: (a) head phantom; (b) body phantom scans. Note that different bowtie filters were employed for each protocol, and that source‐to‐isocenter distance was 100 cm.

In the BEAMnrc simulations, the number of histories was set to two billion for both head and body scan to achieve less than 1% statistical uncertainty in the photon and electron fluence results. A phase space file – which stores particle information such as energy, direction cosines and interaction histories – was acquired for each full‐bowtie and half‐bowtie scan at source‐to‐surface distance (SSD)=80 cm. Thus, the distance from the phase space plane to isocenter was 20 cm, large enough for the body CT phantom whose radius was 16 cm (Fig. 2).

To increase the efficiency of the X‐ray tube simulations, we used a variance reduction technique called Directional Bremsstrahlung Splitting (DBS) with a splitting number of 10,000 (referred from Mainegra‐Hing and Kawrakow's study.^(17)^) We also turned on following “low energy physics options” for accurate simulations: electron impact ionization, bound Compton scattering, photoelectron angular sampling, Rayleigh scattering, atomic relaxation and simple bremsstrahlung angular sampling. National Institute of Standards and Technology (NIST) data were used for the bremsstrahlung cross sections^(^ ^18^ ^,^ ^19^ ^)^ and XCOM data^(^ ^20^ ^,^ ^21^ ^)^ were used for photo‐absorption and Rayleigh scattering cross sections. An electron splitting method was also used to prevent high‐weighted electrons from interacting with other tube component's materials. Both electrons and photons were tracked down to the threshold energy of 1 keV. To create the material data for the 1 keV cutoff energies, we reproduced all the relevant material data by using PEGS4 user code.^(22)^

Two extended MC phantoms were generated to simulate the dose distributions in the CT phantom without losing the long tail portions in the CT dose profiles, shown in Fig. 2. The polymethyl‐methacrylate (PMMA) head/body phantoms (physical density, $\rho =1.19{\text{gcm}}^{-3}$, 16 cm diameter for head and 32 cm diameter for body, 15.2 cm in length) were CT scanned by GE LightSpeed RT 4 scanner (GE Medical Systems, Milwaukee, WI) and the CT DICOM files were used to create two extended MC phantoms (60 cm long for head and 90 cm long for body). These lengths were determined from the results of several trial MC simulations, set to cover the entire dose profile. These extended MC phantoms enabled us to overcome the limitation of the physical CT phantom length. When creating an extended phantom, a single representative slice of the CT DICOM file was imported into the MATLAB system (The MathWorks, Natick, MA) and duplicated the slice into enough number of slices to compose the longer MC phantom. Then, the DICOM header information of each duplicated slice was modified to align the phantoms with the location of the slice. With those duplicated DICOM data, both extended head and body MC phantoms were generated using the CTcreate user code.^(15)^

After the BEAMnrc simulation was finished, the phase space file obtained from the BEAMnrc simulation was re‐used as an input source in DOSXYZnrc^(23)^ simulations to calculate the absorbed dose in the extended MC phantoms. For the rotational irradiation simulation, we used the source type 8 (phase‐space source from multiple directions) with 660 projections, which was the average number of projections of our OBI system. The number of histories was set to 20 billion, which produced less than 1% statistical uncertainties of the photon fluence. Because the MC simulations produce the absorbed dose per an incident particle, a normalization factor was used to correlate the MC results to physical measurements. We employed a calculation method using the technique noted in a previous study.^(24)^

After the CBCT simulations, axial dose profiles at the center and peripheral locations were obtained by using the STATDOSE user code.^(25)^ Subsequently, the absorbed doses on the dose profile were integrated to derive the DPI per each location by Eq. 5:

(5) $$DPI_{l}=\int_{-l/2mm}^{l/2mm}D(z)dz$$

where *l* equals range of longitudinal profile and *D(z)* equals dose profile along the axis of rotation.

Similarly, the cone‐beam CTDI for each beam width was calculated with Eq. 6:

(6) $$CTDI_{a,l}=\frac{1}{T}\int_{-l/2mm}^{l/2mm}D(z)dz=\frac{1}{T}DPI_{l}$$

where *T* equals actual beam width, *l* equals range of longitudinal profile, and *z* equals longitudinal location of profile measurement.

Note that above ${\text{CTDI}}_{a,l}$ equations use the actual beam width T, not the nominal beam width, at the divisor. In the ${\text{CTDI}}_{a,l}$ calculation, both central and peripheral dose profiles were integrated over the range of 600 mm for head scan and 900 mm for body scan in the extended CT phantoms. Similar to the conventional CT system, we also introduced weighted CTDI for CBCT $\left({\text{CTDI}}_{w,l}\right)$ that can represent the volumetric average of the ${\text{CTDI}}_{a,l}$ in the CT phantoms. The ${\text{CTDI}}_{w,l}$ for each beam width was estimated by Eq. 7:

(7) $$CTDI_{w,l}=\frac{1}{2}CTDI_{a,l}^{center}+\frac{1}{2}{\bar{CTDI}}_{a,l}^{periphery}$$

where *l* equals range of longitudinal profile in mm, ${\text{CTDI}}_{a,l}^{\text{center}}$ equals ${\text{CTDI}}_{a,l}$ at a central axis, $\bar{{\text{CTDI}}_{a,l}^{\text{periphery}}}$ equals averaged ${\text{CTDI}}_{a,l}$ at four peripheral locations.

The ${\text{CTDI}}_{a,600}$ for head scan and ${\text{CTDI}}_{a,900}$ for body scan were measured at the center and four peripheral locations which were located at 1 cm below the surface of the CT phantoms. Note that the numerical coefficient 1/2 was chosen to more accurately estimate the ${\text{CTDI}}_{w,l}$ by following Bakalyar's study,^(26)^ which applied a “plausible relative function variation” to the CTDI estimation (as described in the report of AAPM task group 111.^(27)^) The mean ${\text{CTDI}}_{w,l}(\bar{{\text{CTDI}}_{w,l}})$ was calculated by averaging all the ${\text{CTDI}}_{w,l}$ values of various beam widths (1–10 cm nominal beam width). This $\bar{{\text{CTDI}}_{w,l}}$, similar to the Dixon's equilibrium dose parameter $A_{\text{eq}}$, can be interpreted as a single representative dose index for a certain CBCT scan protocol. Finally, we calculated the dose length product for CBCT (DLP) as follows:

(8) $$DLP=\bar{CTDI_{w,l}}\times T$$

where $\bar{{\text{CTDI}}_{w,l}}$ equals averaged ${\text{CTDI}}_{w,l}$ for various beam widths and *T* equals actual beam width.

For the situation when MC simulations are not feasible (i.e., clinical environment), we further investigated accuracy of the CTDI estimation using the point dose measurement. A central ray dose f(0) at the center and four peripheral locations obtained from a previous study^(14)^ were used to calculate the ${\text{CTDI}}_{w}$ (a point dose f(0) in 15.2 cm long standard CT phantoms from ion chamber measurements for a clinical beam width of 20.6 cm) and compared to the above $\bar{{\text{CTDI}}_{w,l}}$ (full integration in extended 60 and 90 cm CT phantoms from MC simulation).

## III. RESULTS

### A. Measured beam profiles

**Figure 3:**
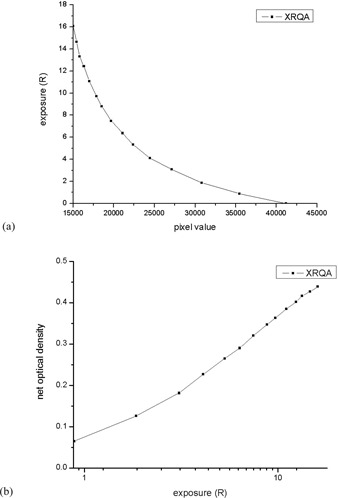
Results of the radiochromic film calibration: (a) calibration curve as pixel value vs. exposure; (b) calibration curve as exposure vs. optical density. Note that the x‐axis in Fig. 3(b) is on the logarithmic scale (log 10).

The calibration curves of radiochromic film are presented in Fig. 3. As can be seen, the PV to exposure graph shows an inverse relationship and the exposure to net optical density (NOD) is relatively linear, as expected. Note that the x‐axis of Fig. 3(b) is in logarithmic scale (log 10).

**Figure 4:**
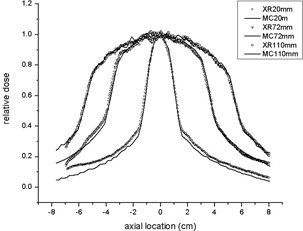
Comparison of axial dose profiles between film measurements and MC simulations. Note that all the profiles were normalized to unity using their central dose values.

The film measured beam width was wider than nominal beam width due to the fact that the CBCT system collimates 1–3 cm wider than the CBCT reconstruction image size. The wider beam widths were expected. The actual beam widths shown in the CBCT system console were as follows: 2.0, 3.4, 4.6, 6.0, 7.2, 8.4, 9.8, 11.0, 12.4, and 13.6 cm. These console‐output data were well matched with the beam widths measured free‐in‐air by the film. The axial dose profiles in the CT phantom from the radiochromic film measurements and MC simulations are presented in Fig 4. As can be seen, the profiles of both methods are relatively well matched, except at the tail portions caused by the lack of photon statistics.

### B. Estimated CTDI and DLP for CBCT

Mean ${\text{CTDI}}_{w,l}$ values, $\bar{{\text{CTDI}}_{w,600}}$ and $\bar{{\text{CTDI}}_{w,900}}$ were found to be 8.74±0.01 cGy for head scan and 4.26±0.01 cGy for body scan, respectively. Note the small standard deviations for both scans which represent the constancy of the ${\text{CTDI}}_{w,l}$ per various beam width. Table 1 presents ${\text{DPI}}^{l},{\text{CTDI}}_{w,l}$, DLP, and central ray dose f(0) values per each beam collimation for both head and body scans acquired from MC simulations in this study and ${\text{CTDI}}_{w}$, mean peripheral dose and f(0) values from measurements and MC results in previous study.^(14)^ One can notice that ${\text{DPI}}_{l}$ increase proportionally, while ${\text{CTDI}}_{w,l}$ is relatively constant over the various beam widths, and f(0) increase monotonically as the beam width increases. Also note that ${\text{DPI}}_{l}$ values are quite close to DLP values because of the same physical definition of both quantities except the use of different ${\text{CTDI}}_{w,l}$ values. ${\text{DPI}}_{l}$ uses individual $\bar{{\text{CTDI}}_{w,l}}$ values while DLP uses the $\bar{{\text{CTDI}}_{w,l}}$ value in each calculation, and both ${\text{CTDI}}_{w,l}$ and $\bar{{\text{CTDI}}_{w,l}}$ are found to be quite close regardless of individual beam width.

**Table 1:** ${\text{DPI}}_{l},{\text{CTDI}}_{w,l}$, DLP and central ray dose f(0) per various beam collimations in head and body CBCT scans from the MC simulations in this study, and ${\text{CTDI}}_{w}$, mean peripheral dose and f(0) from measurements and MC results in previous study.^(14)^ Note that the mean ${\text{CTDI}}_{w,l}(\bar{{\text{CTDI}}_{w}})$ values, obtained in this study, are quite close to the ${\text{CTDI}}_{w}$ values of this and previous studies.

*MC Results in this Study Full‐fan CBCT Scan (Head)*					*MC Results in this Study Half‐fan CBCT Scan (Body)*			
*Beam Width (mm)*	${\text{DPI}}_{600}$ (cGy cm)	${\text{CTDI}}_{w,600}$ (cGy)	DLP (cGy cm)	f(0) (cGy)	Beam Width (mm)	${\text{DPI}}_{900}$ (cGy cm)	${\text{CTDI}}_{w,900}$ (cGy)	DLP (cGy cm)	*f(0) (cGy)*
20	17.48	8.73	17.48	3.68	22	9.38	4.26	9.37	0.84
34	29.72	8.75	29.72	4.68	38	16.20	4.26	16.19	1.17
46	40.21	8.75	40.20	5.32	52	22.16	4.26	22.15	1.44
60	52.45	8.75	52.44	5.96	66	28.13	4.27	28.12	1.67
72	62.94	8.75	62.93	6.44	80	34.10	4.27	34.08	1.88
84	73.43	8.74	73.42	6.82	96	40.92	4.26	40.90	2.08
98	85.66	8.75	85.65	7.20	110	46.88	4.27	46.86	2.27
110	96.15	8.74	96.14	7.49	124	52.85	4.26	52.82	2.39
124	108.39	8.73	108.38	7.74	138	58.82	4.26	58.79	2.55
136	118.88	8.72	118.86	7.99	154	65.64	4.25	65.60	2.65
Mean ${\text{CTDI}}_{w,600}$		8.74	‐	‐	Mean ${\text{CTDI}}_{w,900}$		4.26	‐	‐
			*Measurements and MC Results in Previous Study* ^(14)^						
	*Full‐fan CBCT Scan (Head)*					*Half‐fan CBCT Scan (Body)*			
*Beam Width (mm)*	${\text{CTDI}}_{w}$ (cGy)	Mean peripheral dose (cGy)		f(0) (cGy)		${\text{CTDI}}_{w}$ (cGy)	Mean peripheral dose (cGy)		*f(0) (cGy)*
206	8.50	8.21		8.78	Measured	4.21	5.32		3.09
206	8.77	8.76		8.77	MC	4.26	5.44		3.08

**Figure 5:**
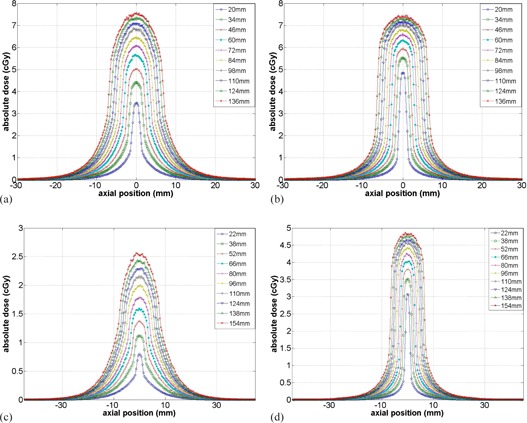
Axial dose profiles of various beam widths at the (a) center and (b) 12 o'clock locations for the head scans, and at the (c) center and (d) 12 o'clock locations for the body scans.

The axial dose profiles of various beam widths for the head and body scans from MC simulations are presented in Fig. 5. The dose profiles at the center (Fig. 5(a) and (c)) were broader than those at the periphery (Fig. 5(b) and (d)), similar to the results of Mori et al.^(8)^ Note that the central ray dose, f(0) in Fig. 5, increases more slowly as the beam width proportionally increases; as the beam width increases, less scattered photon will reach to the center and smaller dose contribution will be made to the central ray dose. It can be also noticed that no heel effect is evident in Fig. 5 because the anode axis of the OBI system is perpendicular to the axis of rotation; the heel effect is smeared out by rotation of the CBCT scan.

For the clinical beam width of 20.6 cm, the f(0) values of the point dose measurements obtained in the previous study^(14)^ were 8.50 cGy (head) and 4.21 cGy (body), and those of the MC simulations were 8.77 cGy (head) and 4.26 cGy (body). Note that these values are recalculated from Table 1 in the previous paper^(14)^ by using the Bakalyar's numerical coefficients, 1/2 and 1/2. The differences between two methods were 3.2% for head scan and 1.2% for body scan.

## IV. DISCUSSION

The dosimetry for the cone‐beam geometry has become more important due to its widespread applications in linac‐mounted or stand‐alone CBCT systems for image‐guided radiation therapy (IGRT), C‐arm interventional CBCT and multislice CT scanners (256 and 320 slices). However, there is no consensus regarding how to evaluate the radiation dose for the CBCT systems; some researchers have adopted the CTDI as a dose descriptor, while others have performed the absorbed dose calculation directly using MC simulations. Although the latter method can provide more accurate dose information such as 3D dose distribution, it requires extensive work that is impractical in clinical environments. In addition, heavy computation time is another disadvantage of MC method.

Recently, Dixon and Boone^(1)^ proposed that the central ray dose f(0), which represents a peak dose, can be a dose descriptor for the CBCT scan. However, this quantity can not be correlated to the radiological risk measure called “effective dose (ED)” because it is just a maximum point dose not an average dose like CTDI. In addition, it cannot be used to estimate the total dose, like DLP can. For this reason, we focused on CTDI, not on f(0), to develop a method of CBCT dosimetry. Once we find the CTDI‐like quantity for a CBCT scan, which is conceptually equivalent to MDCT scan, DLP can be easily derived by this and then ED can be estimated by multiplying a conversion factor to the DLP value. This procedure is quite similar to the ED calculation method in MDCT (ED=conversion factor×DLP).

In this study, we validated a MC method by comparing the CBCT beam profiles obtained from radiochromic film measurements to the MC simulations. Using the MC model, we were able to accurately estimate the CTDI and DLP for a Varian OBI clinical CBCT system. Although we used different numerical coefficients (1/2 and 1/2) in CTDI estimations compared to the studies of Song et al.^(28)^ and Kim et al.,^(14)^ we found that our results are close enough to their data, as shown in Table 1. This MC approach removed the technical limitation of the 100 mm pencil ion chamber measurements in the CTDI estimation in CBCT.

To avoid the limitation of physical measurements, the point dose (central ray dose, f(0)) measurement is another alternative approach to estimate the CTDI in cone‐beam geometry using a small ion chamber and standard CT phantoms. In this study, we investigated the accuracy of the point dose measurement by comparing the measured CTDI values in the standard CT phantoms to the result from the MC simulations (full integration in extended CT phantoms). We found that point dose approach could yield a reasonable accuracy (within 3%) in CTDI estimation for a clinical beam width of 20.6 cm. Thus, the point dose measurement is a clinically useful tool in estimating CTDI of a CBCT system when the MC approach is not feasible.

As aforementioned, it should be emphasized that CTDI and DLP methods only provide an estimation of average absorbed dose to a local body section (head and body), not individual organ doses. Thus, they are not appropriate tools to directly assess the radiation risk of the CBCT. One may be able to derive a conversion factor to relate DLP to ED, but this process inevitably requires an MC simulation with real patients or anthropomorphic phantom geometries in order to obtain the individual organ doses first.^(29)^

This study has a limitation. We employed only one specific CBCT system (Varian OBI model) since it is the only model available in our institution. Other CBCT systems may produce different results. Further investigation will be helpful to understand the applicability of CTDI and DLP to other CBCT systems. In addition, a future study to evaluate the conversion factor between DLP and ED will be of great interest.

## V. CONCLUSIONS

We applied the CTDI concept to CBCT dosimetry, validated the applicability of CTDI as a dose index for CBCT, and incorporated the CTDI with beam collimation to estimate the total CBCT scan dose (DLP). Using this strategy, we successfully estimate the CTDI and DLP for a commercial CBCT system. We expect that these CTDI and DLP values are useful to clinical physicists when they estimate the CBCT dose for the CBCT system. From this DLP value, it will be possible to derive the effective dose (ED) by using a conversion factor which can be obtained from MC simulations. In this study, we also demonstrated a clinically feasible approach to estimate the CBCT dose (CTDI) using the point dose method and showed that the CTDI accuracy of the point dose approach is within 3% compared to the full integration MC method. This point dose approach can be readily applied to other cone‐beam or multi‐detector CT (MDCT) systems.

## ACKNOWLEDGMENTS

We thank Mr. Blake Walters of National Research Council of Canada for discussion regarding BEAMnrc simulations.

## Supporting information

Supplementary Material Files

Supplementary Material Files
