# Supplementary figures and images for: Computed tomography dose index and dose length product for cone‐beam CT: Monte Carlo simulations of a commercial system
Source: J Appl Clin Med Phys. 2011 Jan 19;12(2):84–95. doi: 10.1120/jacmp.v12i2.3395 (PMC5718669; doi:10.1120/jacmp.v12i2.3395)

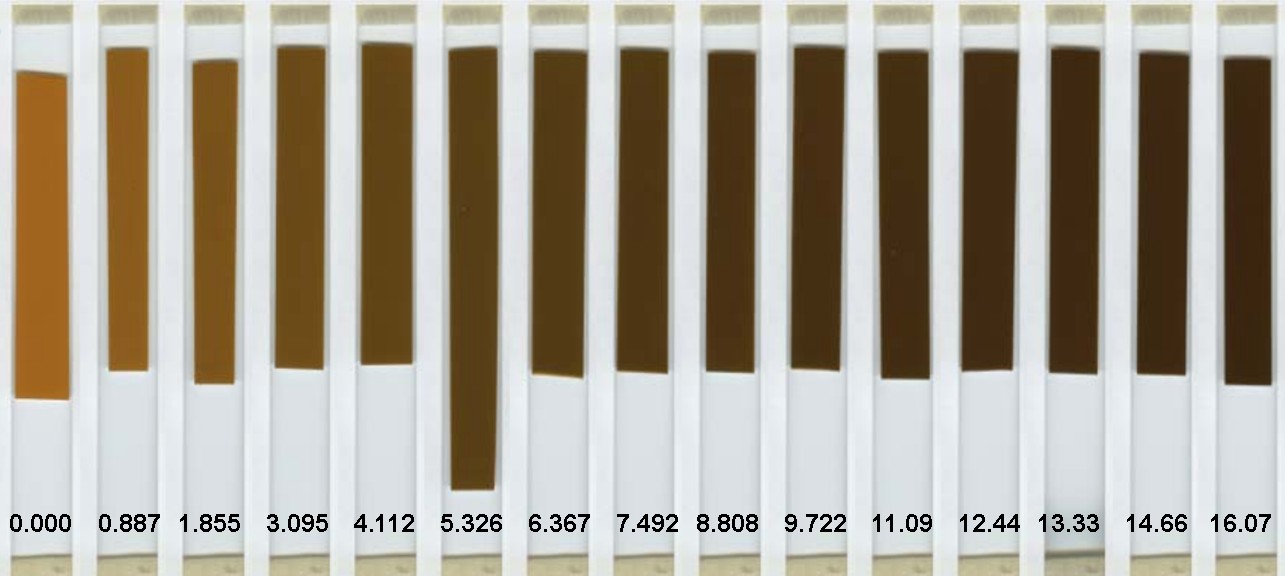

Supplement: Supplementary file 1 — Supplementary Material Files [file ACM2-12-084-s001.jpg]

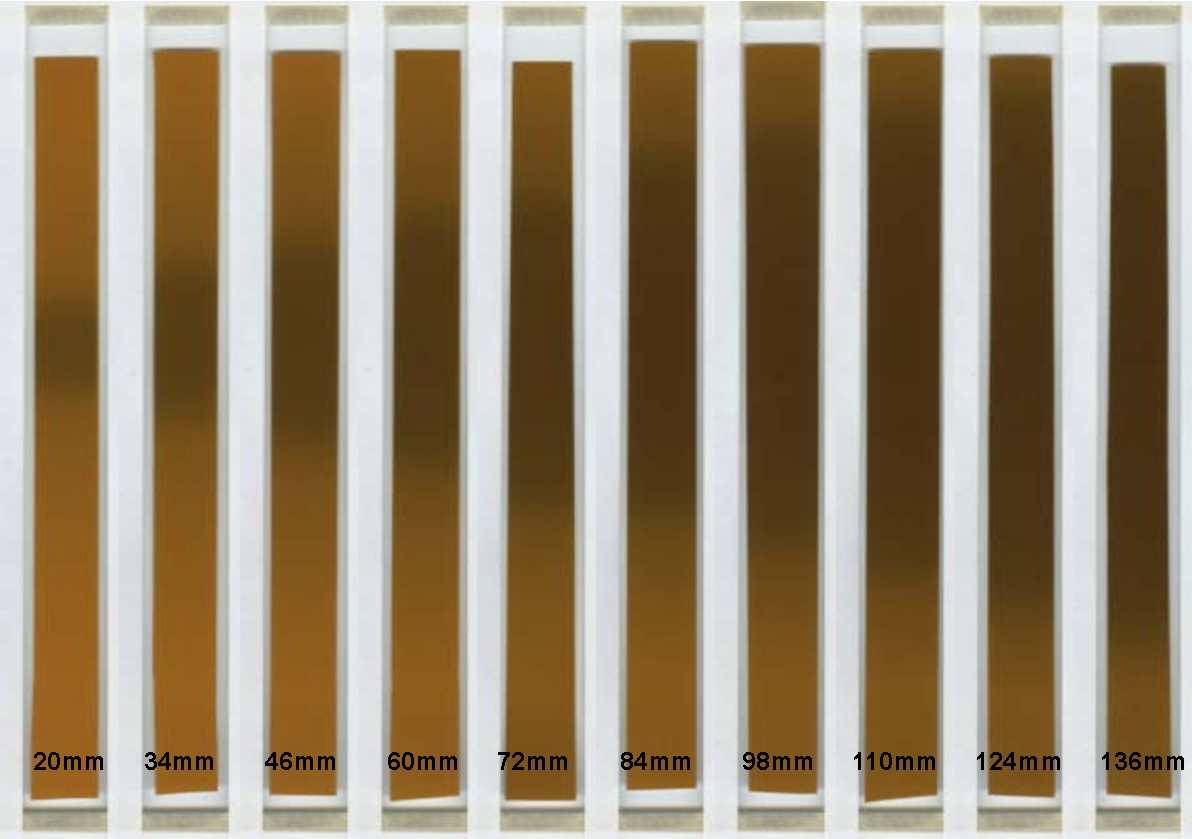

Supplement: Supplementary file 2 — Supplementary Material Files [file ACM2-12-084-s002.jpg]
